# Supplementary material for: TLR4 Asp299Gly (rs4986790) polymorphism and coronary artery disease: a meta-analysis
Source: PeerJ. 2015 Nov 26;3:e1412. doi: 10.7717/peerj.1412 (PMC4671173; doi:10.7717/peerj.1412)
Supplement: Article S1 [file peerj-03-1412-s001.pdf]

Supplemental Article S1

Association between Asp299Gly and the number of stenotic coronary arteries

Study characteristic. A total of five reports (*Boekholdt et al., 2003; Yang, Holloway & Ye, 2003; Hernesniemia et al., 2006; Džumhur et al., 2012; Guven et al., 2015*) were included, and NOS score of all studies was more than 6. By comparing the different genotype distribution in single-vessel, double-vessel, triple-vessel CAD with > 50% stenosis, the association between 299Gly and the severity of CAD could be speculated. Thus, we used single-vessel + double-vessel CAD vs. triple-vessel CAD to clarify the association of the frequency of AA and AG/GG with the number of stenotic vessels.
Meta-analysis results. P_Q = 0.95, I^2 = 0.00%, fixed-effects model was used; OR = 0.93, 95% CI = (0.69, 1.26), *P* = 0.64. Egger test: *P* = 0.08.

No significant difference was found between the genotype frequencies and the number of coronary arteries with > 50% stenosis. The main characteristics of the studies are listed in Table S1. Forest and funnel plots are shown in Fig. S1.

Table S1: Characteristics of studies about the association between Asp299Gly and the number of stenotic coronary arteries.

Study	AA (number of stenosis vessels)			AG+GG (number of stenosis vessels)		
	1	2	3	1	2	3
Yang,2003	393	319	254	48	34	30
Dzumhur,2012	26	24	31	7	3	4
Guven,2015	45	42	53	5	2	3
Boekholdt,2003	249	195	131	32	29	17
Hernesniemi,2006	*	164	61	*	29	9

*Due to the raw data of the study “J. Hernesniemi, 2006”, we could only got the AA/AG+GG genotype frequencies of 1-vessel+2-vessel CAD.

A

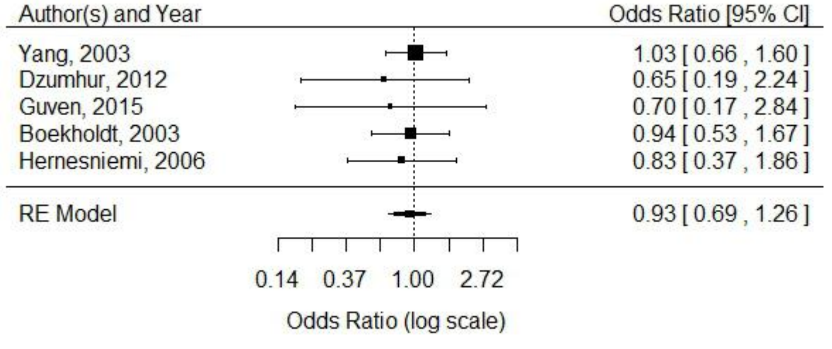

B

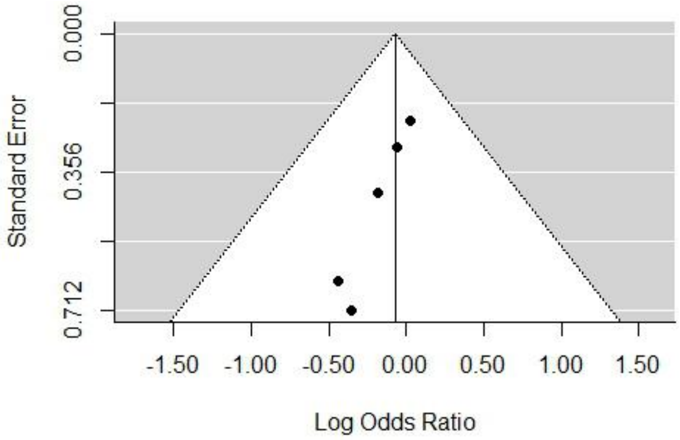

Figure S1: Forest and Funnel plots of the association between TLR4 gene Asp299Gly polymorphism and the number of stenotic coronary arteries. A and B (Forest and Funnel plots for 1-vessel+2-vessel vs. 3-vessel); C and D (Forest and Funnel plots for 1-vessel+2-vessel+3-vessel vs. 3-vessel).
