# Supplementary material for: TLR4 Asp299Gly (rs4986790) polymorphism and coronary artery disease: a meta-analysis
Source: PeerJ. 2015 Nov 26;3:e1412. doi: 10.7717/peerj.1412 (PMC4671173; doi:10.7717/peerj.1412)
Supplement: Article S2 [file peerj-03-1412-s002.pdf]

Supplemental Article S2

Synergistic effect of statin and Asp299Gly

Study characteristics. Boekholdt et al. (*Boekholdt et al., 2003*) was the first to demonstrate that 299Gly modified the efficacy of statin in preventing cardiovascular events, such that carriers of the variant allele had significantly more benefit from statin treatment. However, other reports found no association between 299Gly carriers and the incidence of cardiovascular events in statin-treated patients (*Kolek et al., 2004; Beijk et al., 2010*). The incidence of cardiovascular events according to TLR4 genotype and statin is shown in Table S2. Two groups (statin_AA v.s statin_AG/GG, no statin_AA v.s no statin_AG/GG) were used to clarify the synergistic effect of statin and 299Gly.

Meta-analysis results. statin_AA v.s statin_AG/GG: $P_Q = 0.05$, $I^2 = 67.45\%$, random-effects model was used; OR =1.12, 95% CI = (0.41, 3.09), p = 0.82. Egger test: p = 1.00.

no statin_AA v.s no statin_AG/GG: $P_Q = 0.14$, $I^2 = 48.82\%$, fixed-effects model was used; OR = 0.80, 95% CI = (0.51, 1.25), p = 0.33. Egger test: p = 1.00.

No synergistic effect of statin and Asp299Gly was found. Because of the low number of articles included and large heterogeneity, the result should be confirmed by more high-quality studies in future. Forest plots for the incidence of cardiovascular events according to TLR4 genotype and statin are shown in Fig. S2.

Table S2: Characteristics of studies about the incidence of cardiovascular events according to TLR4 genotype and statin.

Study	Statin				No-Statin			
	AA		AG/GG		AA		AG/GG	
	I	T	I	T	I	T	I	T
Boekholdt,2003	32	278	1	51	54	299	8	27
Kolek,2004	61	285	8	22	391	1437	37	147
Beijk,2010	134	1285	16	176	90	1059	20	162

I: Incidence; T: Total.

A

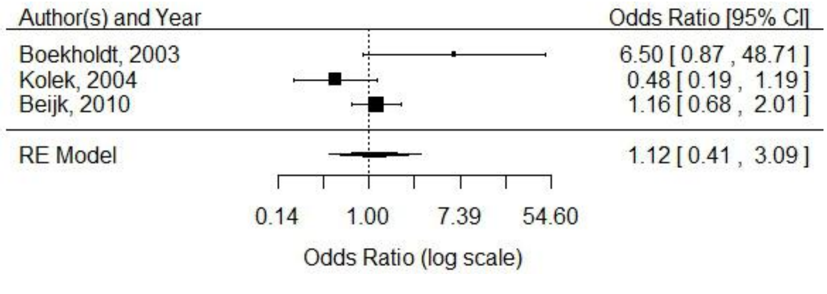

B

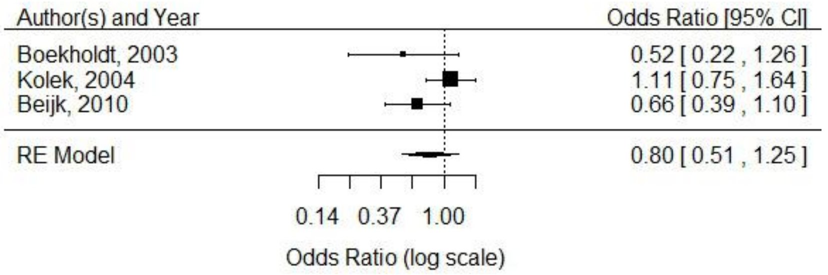

Figure S2: Forest plots for the incidence of cardiovascular events according to TLR4 genotype and statin treatment. A (Statin_AA v.s Statin_AG/GG); B (No Statin_AA v.s No Statin_AG/GG).
